# Arginine vasopressin deficiency after surgical ligation of an unruptured anterior communicating artery aneurysm – a case report and literature review

**DOI:** 10.3389/fendo.2025.1662205

**Published:** 2025-11-18

**Authors:** Julia K. Gundersen, Anne Katrin Torsheim Holmøy, Per Kristian Eide, Jacob Andreas Winther

**Affiliations:** 1 Department of Physiology, Institute of Basic Medical Sciences, University of Oslo, Oslo, Norway; 2 Department of Neurology, Akershus University Hospital, Lørenskog, Norway; 3 Department of Neurosurgery, Oslo University Hospital - Rikshospitalet, Oslo, Norway; 4 Institute of Clinical Medicine, Faculty of Medicine, University of Oslo, Oslo, Norway; 5 KG Jebsen Centre for Brain Fluid Research, University of Oslo, Oslo, Norway; 6 Department of Endocrinology, Akershus University Hospital, Lørenskog, Norway

**Keywords:** vasopressin, insipidus, sodium, osmolality, aneurysm, ACOM, hypothalamus, MRI

## Abstract

**Introduction:**

Plasma osmolality is maintained within a narrow range by secretion of arginine vasopressin (AVP). AVP deficiency (formerly known as central diabetes insipidus) is a rare complication of surgery in the hypothalamic and pituitary region.

**Case description:**

A 50-year-old male patient developed postoperative dehydration, polyuria and cognitive impairment following surgical clip ligation of an unruptured anterior communicating artery (ACOM) aneurysm. Endocrine tests revealed moderate hypernatremia, plasma hyperosmolality and urine hypoosmolality. Magnetic resonance imaging of the brain uncovered delayed cerebral infarctions in genu rostrum of corpus callosum and bilateral oedema of fornix anterior columns. All clinical findings supported the diagnosis of central AVP deficiency, likely involving the anterior hypothalamic nuclei. Follow-up 2 months postoperatively confirmed persisting AVP deficiency and cognitive impairment.

**Conclusion:**

AVP deficiency without associated haemorrhage is exceedingly rare, with only seven cases identified in our literature review. This case highlights that postoperative AVP deficiency may present with mild or unspecific symptoms. Importantly, concurrent cognitive impairment may occur, further complicating compliance to treatment. Therefore, close postoperative monitoring is crucial for correct diagnosis and bed-side management.

## 1 Introduction

Sodium is a major determinant of osmotic pressure; hypernatremia is therefore a common cause of hyperosmolality. Clinically, subacute plasma hyperosmolality may manifest as confusion, dysphoria, drowsiness, convulsions or coma (1). In acute cases, it may cause substantial brain volume loss (2), increasing the risk of haemorrhage and irreversible brain injury (1). The body is able to maintain plasma osmolality within a narrow physiological range via the hormone arginine vasopressin (AVP) (3). AVP is released in response to extracellular hyperosmolality and maintains homeostasis by regulating renal water reabsorption. AVP is synthetised within the supraoptic nuclei (SON) and the paraventricular nuclei (PVN) of the anterior hypothalamus, transported along the neuronal axons and released from the neurohypophysis (4). AVP-release is triggered by specialised osmoreceptors, such as the delta-n transient receptor potential vannilloid (TRPV1) channel, located in the subfornical organ (SFO), organum vasculosum of the lamina terminalis (OVLT) and median preoptic nucleus (MnPO). These neurons also coordinate drinking behaviour (5). TRPV1-activation is primarily driven by changes in cell volume, indicating that osmoreceptors function essentially as mechanical stretch receptors (6). Interestingly, TRPV1 is also involved in sensation of body temperature (6).

AVP deficiency is characterised by polyuria, leading to dehydration and hyperosmolality. Hyperosmolality typically triggers a pronounced thirst response, but damage to the osmoreceptors or the neural circuitry mediating thirst perception can lead to simultaneous adipsia. The most common causes of central AVP deficiency are cerebral pathology or surgical intervention affecting the hypothalamus or pituitary gland. AVP deficiency has been reported in association with intracranial aneurysms, in particular those affecting the anterior communicating artery (ACOM), although involvement of anterior cerebral artery and middle cerebral artery aneurysms has also been documented (7). In the majority of these cases, AVP deficiency occurs following rupture of an ACOM aneurysm and subsequent subarachnoid haemorrhage (SAH) (**Table 1**). However, in very rare instances, a patient may develop AVP deficiency following surgical ligation of an unruptured ACOM aneurysm. Here, we report such a rare clinical case, which presented with non-specific symptoms that can be challenging to recognise and treat in the clinical setting.

**Table 1:** A systematic literature review of AVP deficiency and ACOM aneurysm surgery was performed in PubMed, MEDLINE and Embase.

Publication	Patient	Sex/age	Rupture	Adipsia	Thermo-dysregulation	Cognitive impairment
Spiro and Jenkins 1971 (36)	n=1	F/52	Yes	Yes	Hypothermia	Not reported
Nussey et al., 1986 (37)	n=1	M/30	Yes	Yes	Hypothermia	Impairment of memory and learning
McIver et al., 1991 (38)	n=2	F/39; M30	Yes; Yes	Yes; Yes	No; No	Not reported; Depression
Pearce et al., 1991 (39)	n=1	M/28	Yes	Yes	Hypothermia	Impaired memory
Ball et al., 1997 (40)	n=1	M/28	Yes	Yes	Reported, no specified	Not reported
Nguyen et al., 2001 (41)	n=1	M/41	Yes	Yes	Not reported	Impaired short-term memory
Smith et al., 2002 (8), and Crowley et al., 2007 (10)	n=4	F/39; M/30; M/40; M/28	No	Yes; Yes; Yes; Yes	No; No; No; Hypothermia	Not reported
Lima et al., 2004 (9)	n=1	M/53	No	Yes	Not reported	Impaired memory
Mavrakis et al., 2008 (42)	n=1	F/55	Yes	Yes	Not reported	Impaired short-term memory
Bin Park et al., 2009 (11)	n=1	F/52	No	Yes	–	Impaired memory
Fukuda et al., 2014 (43)	n=1	F/61	Yes	No	Not reported	Impaired memory
Kurihara et al., 2014 (44)	n=1	M/55	Yes	Yes	Not reported	Impaired short-term memory and personality change
Cuesta et al., 2016 (45)	n=1	M/51	Yes	Yes	Not reported	Not reported
Nolan et al., 2016 (16)	n=1	F/36	Yes	Yes	Not reported	Impaired short-term memory
Tan et al., 2016 (12)	n=1	M/52	No	Yes	Not reported	Impaired memory
Imai et al., 2017 (46)	n=1	M/38	Yes	Yes	Not reported	Not reported
Sabzghabaei et al., 2018 (47)	n=1	M/57	Yes	Yes	Not reported	Not reported
Mullaguri et al., 2020 (48)	n=1	F/55	Yes	No	Not reported	Not reported
Kim et al., 2021 (49)	n=1	F/37	Yes	Yes	Not reported	Not reported
Arora et al., 2022 (50)	n=1	F/63	Yes	No	Not reported	Not reported
Rabiei et al., 2022 (51)	n=1	M/46	Yes	Not reported	Not reported	Not reported
de Silva et al., 2023 (52)	n=1	F/33	Yes	Polydipsia	Not reported	Not reported
Barnett et al., 2024 (53)	N=1	M/65	Yes	Yes	Not reported	Not reported

Highlighted rows indicate cases without ACOM aneurysm rupture. A comprehensive overview of the combination of MESH-terms is presented in **Supplementary Table 1**.

To evaluate the rarity of this condition, we also performed a systematic literature review of AVP deficiency following ACOM aneurysm surgery. A comprehensive search using PubMed, Ovid MEDLINE and Embase of papers published between 1^st^ of January 1946 and 29^th^ of May 2025 was performed using the MESH-terms presented in **Supplementary Table 1**. Publications describing cases of AVP deficiency following ruptured or unruptured ACOM aneurysms after surgical ligation were included (**Table 1**). To date, only seven cases of AVP deficiency following unruptured ACOM aneurysm surgery have been reported worldwide (8–12) (**Table 1**), in addition to the present case.

## 2 Case description

### 2.1 Clinical presentation

A 50-year-old man was diagnosed with an unruptured ACOM aneurysm measuring 8 mm in diameter, with no prior history of cerebral bleeding. Significant medical history included granulomatosis with polyangiitis (GPA, formerly known as Wegener’s granulomatosis) with renal involvement, treated with daily prednisolone (5 mg) and annual rituximab infusions, the last of which was administered 6 months prior to surgery.

Given the aneurysmal size of 8 mm, surgical repair was indicated and consensus on repair modality was reached in a multidisciplinary team of interventional radiologists and neurosurgeons. Initially, a stent procedure was favoured due to the wide aneurysmal neck, however, clip ligation was ultimately recommended because of a right hypoplastic A1. The procedure was performed through a right pterional craniotomy under general anaesthesia. The aneurysm was exposed via the Sylvian and interhemispheric fissures without tissue resection. Due to several perforators at the base of the ACOM aneurysm providing collateral perfusion, clipping was done under temporary occlusion of the left A1 and A2 arteries. Micro-Doppler was used to verify open vessels after clipping, particularly the open perforators from the ACOM. Topical papaverine was applied to prevent vasospasms. Postoperatively, no immediate neurological deficits were encountered, but disorientation was noted. Cerebral computed tomography (CT) and angiography (CTA) on the first postoperative day revealed a closed aneurysm with no evidence of vessel occlusions.

Two days postoperatively, the patient was transferred to the department of neurology for postoperative care. Upon admission, he had a Glasgow Coma Scale (GCS) score of 14 due to disorientation, but no other clinical neurological deficits. On the following day he exhibited signs of delirium and somnolence with a GCS score of 13. Over the subsequent days, delirium persisted and was accompanied by increasing restlessness, for which haloperidol was prescribed in addition to standard clinical care. During the first week of admission, ongoing delirium and cognitive impairment prompted a formal cognitive assessment by an occupational therapist. The patient scored 18/30 on the Mini Mental State Examination (MMSE), which is markedly abnormal for his age and substantially deviating from his reported preoperative cognitive status.

**Figure 1:**
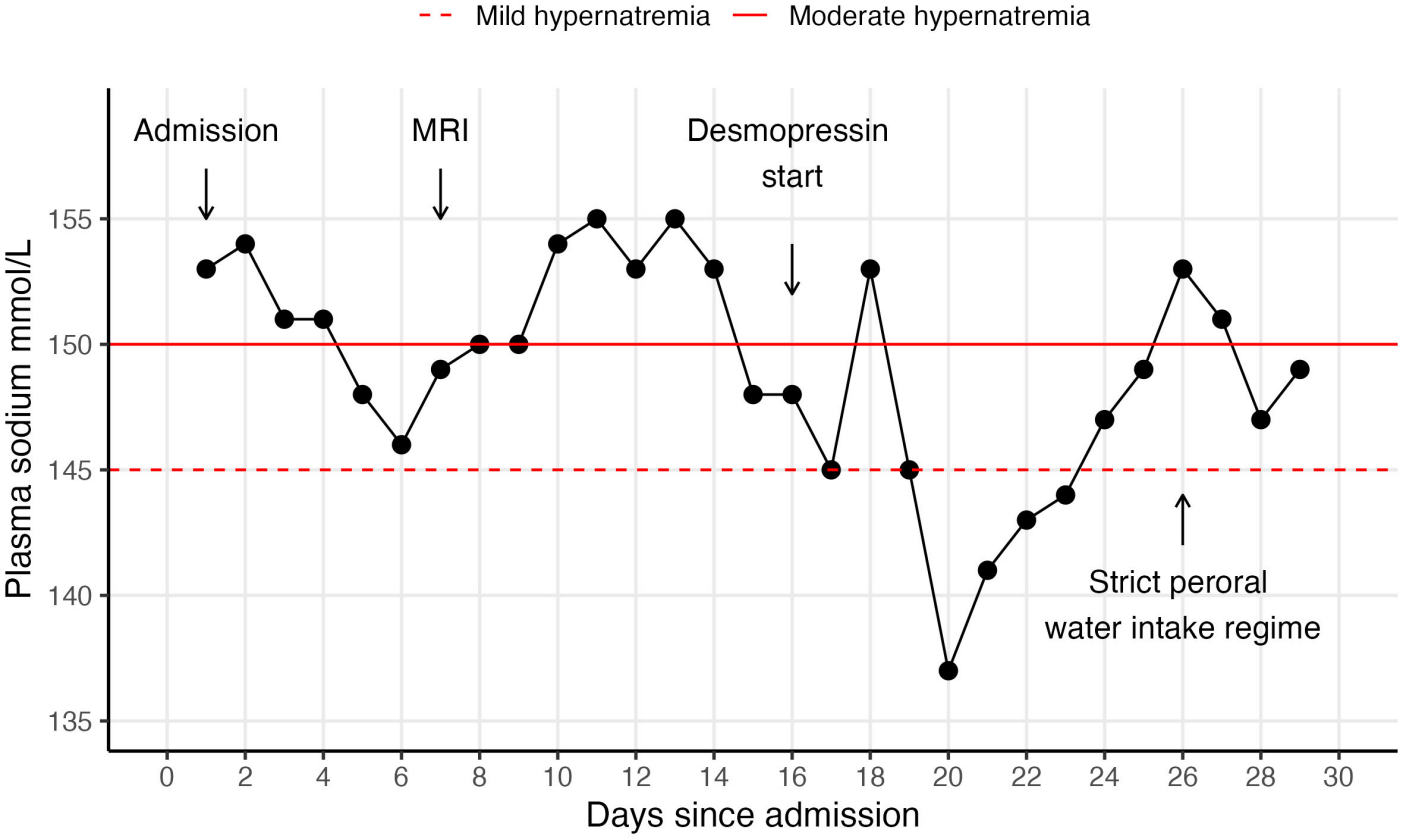
Timeline of daily monitoring of serum sodium levels. The patient was administered desmopressin treatment from day 16 of admission. Daily IV glucose 5% IV infusions were stopped on day 20, after which the plasma sodium concentration gradually increased despite optimal desmopressin dosage due to adipsia. Strict peroral water intake of 2-2.5 L was recommended on day 26, after which the plasma sodium concentration gradually increased due to adipsia despite optimal desmopressin dosage.

The patient’s vital signs were within normal physiological ranges, exception for a low-grade fever during the first few days. Biochemically, he exhibited elevated C-reactive protein (CRP) of 56 mg/L, creatinine of 129 μmol/L, an estimated glomerular filtration rate (eGFR) of 54 mL/min/1.73 m² and marked hypernatremia of 153 mmol/L. The hypernatremia was initially attributed to reduced perioperative water intake and managed with intravenous 5% glucose infusions. While CRP levels normalised over a few days, sodium and creatinine levels remained elevated despite daily administration of 2–4 L of 5% glucose solution (**Figure 1**). He began to exhibit clinical signs of dehydration, such as reduced skin turgor and dry oral mucous membranes, and reported excessive urination. The persistent hypernatremia prompted further evaluation by an endocrinologist. A comprehensive panel of blood and urine tests (**Table 2**) revealed high plasma osmolality (305 mOsm/kg) and hypernatremia (148 mmol/L), combined with low urine osmolality (205 mOsm/kg). These results indicated impaired ability to concentrate urine and conserve water.

**Table 2:** A comprehensive panel of endocrine tests was obtained on day 16 of admission.

Lab test	Units	Value	Reference range
Haemoglobin	[g/dL]	13.7	(13.4 - 17.0)
Sodium	[mmol/L]	148	(137 - 145)
Potassium	[mmol/L]	4.1	(3.6 - 5.0)
Calcium	[mmol/L]	2.25	(2.15 - 2.51)
Magnesium	[mmol/L]	0.81	(0.71 - 0.94)
Creatinine	[μmol/L]	119	(60 - 105)
eGFR	[mL/min/1.73m²]	60	(> 62)
P-osmolality	[mosmol/kgH2O]	305	(280 - 300)
U-osmolality	[mosmol/kgH2O]	205	(> 800)
Copeptin	[pmol/L]	2.1	(1.70 - 11.25)
Prolactine	[mIE/L]	886	(90 - 325)
TSH	[mIE/L]	1.4	(0.30 - 4.2)
T4	[pmol/L]	14.4	(12.0 - 22.0)
FSH	[U/L]	7.3	(1.4 - 12.0)
LH	[U/L]	5.4	(1.7 - 8.6)
Testosterone	[nmol/L]	9.2	(5.3 - 25.0)
ACTH	[pmol/L]	4.0	(1.4 - 14.0)
Cortisol	[nmol/L]	30	(133 - 537)
GH	[μg/L]	0.56	(< 2.50)
IGF-1	[nmol/L]	21	(9.5 - 26.2)
Glucose	[mmol/L]	7.1	(< 11.0)

The deviating values included hypernatremia, renal impairment, plasma hyperosmolality, urine hypoosmolality, hyperprolactinemia and reduced morning cortisol, the latter being attributed to concurrent prednisolone treatment. eGFR, estimated glomerular filtration rate; P, plasma; U, urine; TSH, thyroid stimulating hormone; T4, thyroxine; FSH, follicle stimulating hormone; LH, luteinising hormone; ACTH, adrenocorticotropic hormone; GH, growth hormone; IGF-1, insulin-like growth factor 1.

### 2.2 Diagnostic assessment and therapeutic intervention

An impaired ability to concentrate urine in the setting of elevated plasma osmolality after brain surgery suggested the diagnosis of AVP deficiency, although the typical thirst sensation was absent. A low plasma concentration of copeptin (2.1 pmol/L), the C-terminal segment of the AVP peptide precursor co-secreted in equimolar amounts (13, 14), provided further support for the diagnosis. Finally, administration of desmopressin (a synthetic AVP analogue) resulted in a marked improvement in renal concentration ability, with urine osmolality ultimately reaching 950 mOsm/kg, thereby confirming the diagnosis of AVP deficiency.

**Figure 2:**
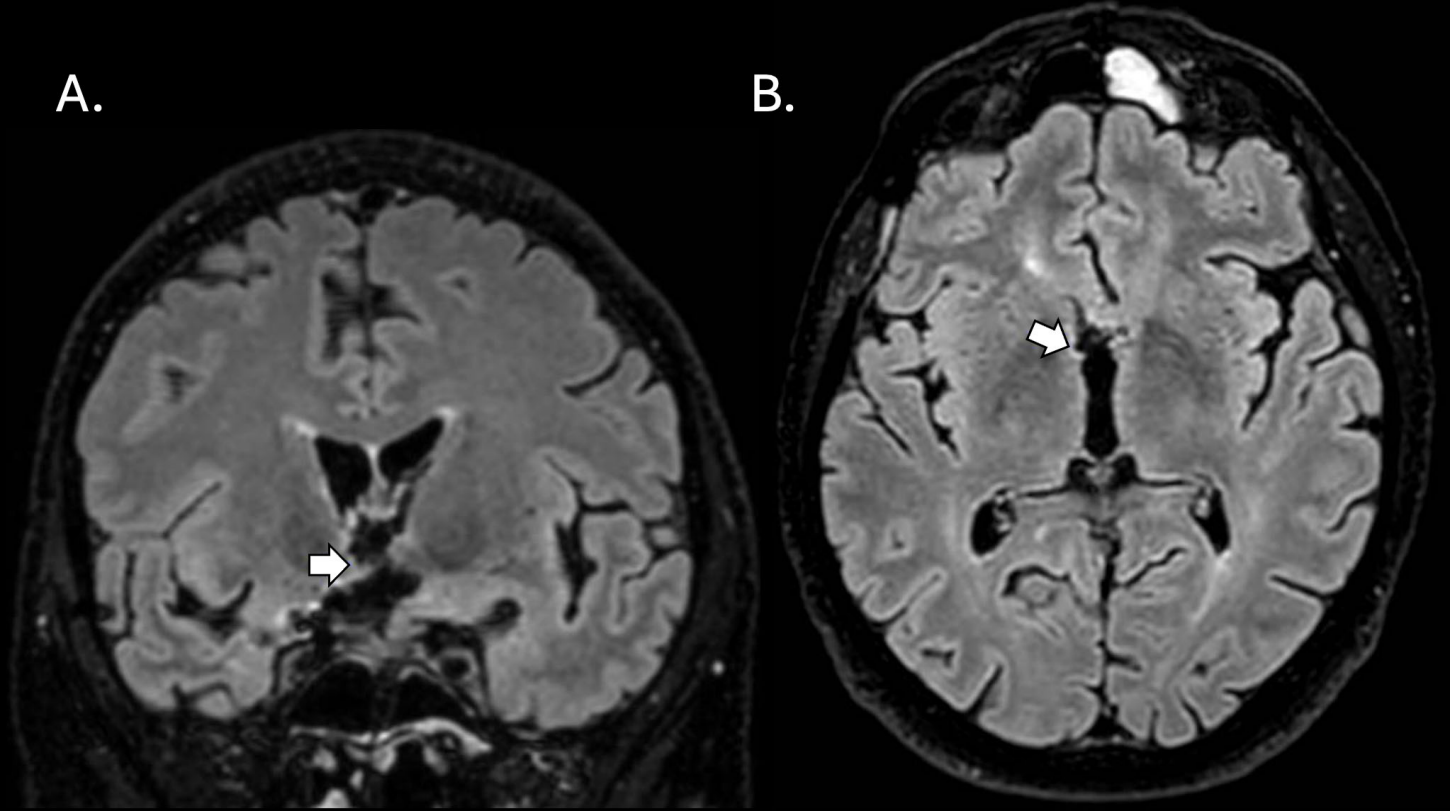
Magnetic resonance imaging (MRI) FLAIR SPIR 1.2mm scans of the brain obtained at follow-up 1 month after the surgical clip ligation. White arrows in coronal **(A)** and axial **(B)** planes point to subacute infarction in the right hypothalamus. MRI scans were assessed by a specialist neuroradiologist (J.O.).

Due to these unexpected findings, a magnetic resonance image (MRI) scan of the brain was performed on the 7^th^ postoperative day (**Figure 2**). The MRI revealed infarction in the genu rostrum of corpus callosum and bilateral oedema of the fornix anterior columns and the hypothalamus, in addition to ischemic changes nearby the surgical route (the anterior temporal and the basal frontal pole). No changes were observed in the hippocampus or the mamillary bodies. A follow-up MRI confirmed the infarctions. An MRI of the pituitary gland was also obtained, which showed no pathology in the pituitary gland.

Despite reaching adequate urine osmolality on desmopressin treatment, maintaining normal plasma sodium levels proved difficult without water supplementation in the form of glucose 5% infusions. The patient did not experience thirst despite dehydration and elevated plasma osmolality, a state that under normal conditions would elicit a strong thirst response. This necessitated a strict fluid regimen to ensure sufficient oral fluid intake before stable water homeostasis could be achieved. He was advice to maintain a daily water intake of 2-2.5 L.

### 2.3 Follow-up and outcome

The patient was scheduled for follow-up assessment with both a specialist neurologist and specialist endocrinologist within 1 month of discharge (approximately 2 months postoperatively). At follow-up, he appeared in good general condition, well-hydrated and did not report fluctuations in weight. His blood analysis showed normal levels of osmolality and sodium concentration. However, the patient still reported difficulty with maintaining an adequate water intake, which he attributed to a lack of thirst and forgetfulness regarding drinking water. Interestingly, he also described a persistent sensation of feeling cold, despite his skin being normothermic to the touch and other household members perceiving the ambient temperature as warm.

Repeated assessment of his cognitive function revealed a decline in selective attention, learning and memory recall. In contrast, executive function, processing speed, and working memory remained within the normal limits. These findings indicate persistent cognitive impairment with amnesia, identifying him as a candidate for further cognitive rehabilitation, which he was scheduled for in the near future.

### 2.4 Patient perspective

The patient and his family were informed of the diagnosis and the likelihood that it represented a postoperative complication. He reported significant impact on his quality of life since discharge, providing the example that he was unable to resume work as usual due to the difficulties posed by his impaired memory. He also emphasised the importance of clinician awareness of this condition in order to reduce the risk of and improve the diagnosis of postoperative AVP deficiency. At the time of his statement, the patient was still undergoing cognitive rehabilitation.

## 3 Discussion

We present a rare case of hypothalamic dysfunction – manifesting as AVP deficiency, adipsia cognitive impairment, and thermodysregulation – following surgical clip ligation of an unruptured ACOM aneurysm. Th clinical picture is consistent with partial hypothalamic syndrome (15) likely secondary to delayed ischemia of the anterior hypothalamus, including the nuclei responsible for osmoregulation. This case highlights the anatomical relationship between the anterior hypothalamus and the ACOM. AVP-producing neurons in the SON and PVN receive blood supply from the perforating branches of the ACOM (16, 17), which may be compromised by vasospasms of the perforators. Similarly, the osmosensitive neurons in the SFO, OVLT and MnPO are supplied by perforators of the anterior cerebral artery and anterior choroid artery (17, 18). Interestingly, excessive AVP secretion is a known complication of subarachnoid haemorrhage, leading to hyponatremia in the syndrome of inappropriate antidiuretic hormone secretion (SIADH) (19–21), for which the ACOM is the most commonly affected site (22).

Delayed cerebral ischemia is commonly associated with ruptured cerebral aneurysms; however it is increasingly recognised as a complication to surgical clip ligation of unruptured cerebral aneurysms as well (23, 24). Prophylactic treatment of vasospasms with calcium-channel blocker nimodipine may be considered, although its efficacy remains to be validated. Ischaemic complications have also been reported following endovascular repair of unruptured cerebral aneurysms (25).

The patient developed persistent cognitive impairment postoperatively, most likely related to ischaemia of the anterior fornix. No visible ischemic lesions in the hippocampus or mammillary bodies were detected. Notably, AVP deficiency has been linked to cognitive impairment in several other case reports (**Table 1**). Experimental evidence indicates that AVP may play a direct role in formation of new memories in the hippocampus (26). Importantly, cognitive impairment may critically impede compliance to desmopressin and appropriate water intake, especially when combined with adipsia. This was the case with our patient. Despite administering high doses of desmopressin, his plasma sodium levels surged when IV fluid infusions were stopped (**Figure 1**), representing inadequate water intake. Also, reliable urine volumes were difficult to obtain due to reduced compliance. It is important for clinicians to recognise that adequate water consumption is just as crucial as desmopressin therapy in the management of patients with AVP deficiency and adipsia.

The patient also exhibited signs of thermodysregulation, manifesting as a persistent sensation of feeling cold. Thermodysregulation, along with endocrine dysfunction and cognitive impairment are recognised features of hypothalamic dysfunction. Increased appetite and sleep disorders are also commonly reported in this context (27), however, our patient did not experience such symptoms. Speculatively, the altered thermoregulation reported by our patient may also be related to dysfunction TRPV1 expressing neurons in the hypothalamus, even in the absence of visible lesions on the MRI. TRPV1 channels are known to play a role in thermoregulation, and experimental studies have shown that TRPV1 agonists (e.g. capsaicin) induce hypothermia that is not reversed by hot ambient temperature (28).

Several factors complicated the interpretation of this case. First, hypernatremia is a common postoperative comorbidity in neurological patients. Given the absence of other neurological symptoms, the patient’s cognitive impairment and/or lethargy could have been explained by transient postoperative hypernatremia. Unfortunately, a reliable assessment of fluid balance, which is a key to diagnosing AVP deficiency, was difficult to obtain due the patient’s persistent cognitive impairment. A second complicating factor was the patient’s co-morbidity with GPA. Autoimmune multisystem diseases, such as GPA may be accompanied with cerebral vasculitis (29), which has been reported to cause subarachnoid (30, 31) and intracerebral (32) haemorrhages, as well as brain infarction (33). GPA thus represents a risk factor for the postoperative complications. In rare cases, GPA may also involve the pituitary gland, causing hypophysitis and dysfunction with AVP deficiency being the most prevalent manifestation (34, 35). However, in this case, the absence of pituitary abnormalities on MRI, along with no visual disturbances, no evidence of other pituitary axes dysfunction, and no multisystem organ involvement (e.g. respiratory system) ultimately weakened the clinical suspicion of autoimmune hypophysitis.

## 4 Conclusion

AVP deficiency without associated SAH is a rare complication to repair of an unruptured ACOM aneurysm. Because patients may exhibit only mild neurological and/or cognitive symptoms, close postoperative monitoring is crucial for timely diagnosis and effective bedside management. Prophylactic treatment with nimodipine may be considered to reduce the risk of vasospasms and delayed cerebral ischemia. Importantly, adipsia in conjuncture with cognitive impairment may significantly reduce adherence to desmopressin therapy and adequate water intake, increasing the risk of developing outpatient hypernatremia. To mitigate this, clear patient education and structured follow-ups are critical to support compliance and prevent complications.

## Data Availability

The datasets presented in this article are not readily available because Clinical blood samples are available in **Table 2**. Any other data or clinical information is considered identifiable, and therefore not available. Requests to access the datasets should be directed to juliakgundersen@hotmail.com.

## Supplementary material

Supplementary Table 1 The systematic literature review collected publications, which were then manually screened based on title, abstract and full text by one investigator (JKG). Duplicate cases reported in multiple papers were excluded, with only the original paper included. Only cases reporting on ACOM pathology or surgery where included. The search was assisted by staff at the medical library at Akershus University Hospital.
